# How Anxiety State Influences Speech Parameters: A Network Analysis Study from a Real Stressed Scenario

**DOI:** 10.3390/brainsci15030262

**Published:** 2025-02-28

**Authors:** Qingyi Wang, Feifei Xu, Xianyang Wang, Shengjun Wu, Lei Ren, Xufeng Liu

**Affiliations:** 1 School of Basic Medicine, Air Force Medical University, Xi’an 710032, China; jussy@126.com (Q.W.); 15102931199@163.com (F.X.); 2 School of Military Medical Psychology, Air Force Medical University, Xi’an 710032, China; wangxianyang_1999@163.com (X.W.); wushj@fmmu.edu.cn (S.W.); 3 Military Psychology Section, Logistics University of PAP, Tianjin 300309, China; 4 Military Mental Health Services & Research Center, Tianjin 300309, China

**Keywords:** anxiety assessment, speech parameters, network analysis, ecological validity, psychological stress

## Abstract

**Background/Objectives**: Voice analysis has shown promise in anxiety assessment, yet traditional approaches examining isolated acoustic features yield inconsistent results. This study aimed to explore the relationship between anxiety states and vocal parameters from a network perspective in ecologically valid settings. **Methods**: A cross-sectional study was conducted with 316 undergraduate students (191 males, 125 females; mean age 20.3 ± 0.85 years) who completed a standardized picture description task while their speech was recorded. Participants were categorized into low-anxiety (n = 119) and high-anxiety (n = 197) groups based on self-reported anxiety ratings. Five acoustic parameters—jitter, fundamental frequency (F0), formant frequencies (F1/F2), intensity, and speech rate—were analyzed using network analysis. **Results**: Network analysis revealed a robust negative relationship between jitter and state anxiety, with jitter as the sole speech parameter consistently linked to state anxiety in the total group. Additionally, higher anxiety levels were associated with a coupling between intensity and F1/F2, whereas the low-anxiety network displayed a sparser organization without intensity and F1/F2 connection. **Conclusions**: Anxiety could be recognized by speech parameter networks in ecological settings. The distinct pattern with the negative jitter-anxiety relationship in the total network and the connection between intensity and F1/2 in high-anxiety states suggest potential speech markers for anxiety assessment. These findings suggest that state anxiety may directly influence jitter and fundamentally restructure the relationships among speech features, highlighting the importance of examining jitter and speech parameter interactions rather than isolated values in speech detection of anxiety.

## 1. Introduction

The rapid pace and high-pressure demands in contemporary society can easily induce emotional responses such as the anxiety state in individuals [1,2]. These negative emotions may cause impaired decision-making [3], reduced work efficiency [4], and strained interpersonal relationships [1], ultimately impacting overall productivity and an individual’s well-being [5]. These emotions are largely driven by the persistent stressors of daily life [2]. Distinct from chronic anxiety disorders, the anxiety state is a transient emotional response that individuals experience when exposed to situational stressors, such as stringent deadlines, performance evaluations, and social pressures [6,7]. Despite this growing prevalence, the methods for its assessment remain subjective and time-consuming [8,9]. Traditional evaluation of anxiety has predominantly relied on self-report questionnaires, clinical interviews, and behavioral observations, which, while providing valuable insights into subjective experiences, are susceptible to reporting biases and recall limitations [10,11]. These conventional approaches are typically supplemented by physiological measurements, including cardiovascular monitoring, blood pressure measures, and cortisol sampling, which offer objective biological markers of stress responses [12,13]. Contemporary wearable devices enable continuous tracking of various physiological parameters, including heart rate variability or electrodermal activity [14]; however, these measurements require direct contact between the sensing elements and the skin surface.

Speech has in recent years arisen as a particularly promising approach for anxiety detection, with its non-invasive quality, remote measurement capability, and potential for continuous monitoring [15,16]. It could reflect psychological states through synchronizing muscles across respiratory, phonatory, and supralaryngeal organs, working in line with cognitive and emotional processing [17,18]. The intricate interplay between these systems makes speech particularly valuable as a biomarker, as it can capture significant motor, cognitive, and behavioral changes associated with mental health conditions [19,20].

Individuals with anxiety disorders exhibit heightened activation of the sympathetic nervous system under stress [21,22], which directly influences speech production through various pathways [23]. This manifests in different features, including speech rates, increased disturbances, and hesitations [8,24,25], which could be measured by speech parameters such as fundamental frequency, formant frequencies, and various spectral properties [9,26]. Most research studies on anxiety have reported increased F0 in anxious individuals [8,24]. Besides F0, parameters such as jitter, shimmer, and pause patterns [9,14] and speech rate [27] reveal further complexity in anxiety-induced vocal expression. Anxiety may further impair articulatory precision, reducing vowel clarity and altering formant bandwidths as a result of stress-induced physiological arousal [28,29,30]. The anxiety state, as a transient response to situational stressors, often correlates with vocal parameters, including elevated fundamental frequency (F0), increased intensity, accelerated speech rate, and decreased duration [9,14,31,32,33].

Studies have been performed on speech parameters in anxiety, highlighting key sensitive indicators including the following: (1) Fundamental frequency (F0), which represents the rate of vocal cord vibration and corresponds to perceived pitch, has been consistently identified as a primary indicator of stress. Multiple studies have demonstrated a reliable increase in F0 under anxious or stressed conditions [15,34]. This elevation in F0 has been attributed to increased muscle tension and respiratory changes during stress responses [35]. (2) Formant frequencies, particularly the first (F1) and second (F2) formants, serve as crucial indicators of vocal tract resonance and articulation patterns. Research has shown that stress-induced changes in muscle tension and breathing patterns can affect formant characteristics [30]. The F1/F2 ratio has emerged as a potentially valuable metric, though individual variations in these parameters suggest speaker-specific patterns rather than universal trends [14,36]. (3) Jitter, which quantifies cycle-to-cycle frequency variation in vocal fold vibration, has demonstrated mixed results in anxiety detection. While some studies report decreased jitter under stress conditions [37], others have found more variable patterns, suggesting that jitter’s relationship with anxiety may be modulated by individual differences and specific stressor types [15]. (4) Intensity, measured as the amplitude or loudness of speech signals, typically shows elevation under an anxious state, reflecting increased subglottal pressure and muscular tension [38]. However, the magnitude of these changes can vary significantly based on the nature and intensity of the stressor. (5) Speech rate, often quantified through mean voiced segment length, indicates temporal features of stress-affected speech production. Stress typically leads to alterations in speaking patterns, including pause and segment duration [35]. Additionally, spectral features (MFCC, LPCC) and the harmonics-to-noise ratio (HNR) also tap into the acoustic complexity associated with stress, yet HNR sensitivity appears more consistent in physical stress (e.g., workouts) than in tasks involving heavy cognitive load or psychological stress [37,38,39,40]. Overall, these findings support the notion that anxiety-driven physiological arousal alters speech production through multiple, interrelated pathways, positioning acoustic features as viable indicators of psychological stress in various contexts.

To date, much of the existing research on anxious speech has been conducted in controlled laboratory settings, with limited ecological validity in the findings [28]. Real-world scenarios, such as those involving social evaluation or time pressure, presumably would be more ecologically valid for investigating anxiety-related speech changes. Moreover, previous studies have primarily focused on isolated speech parameters, overlooking the complex interrelations between these features [25,41]. For example, while F0 and jitter are often studied independently, their interactions with formants, intensity, and speech rate under anxiety state are poorly understood.

Network analysis, which is one of the state-of-the-art tools to identify and analyze the pattern of statistical association in multivariate data, has achieved exponential development in the field of psychological science [42]. Networks include nodes and edges. Nodes represent the variables of study and edges represent connections between nodes. Compared with traditional statistical models, network analysis offers the following methodological advantages for investigating the relationships among anxiety state and related speech parameters: (1) Visualization of systemic dynamics. It illustrates direct edges, indirect pathways, and central nodes in a unified framework, mitigating the reductionism and isolated focus of traditional methods [43]. (2) Group-level network comparison. Rather than treating parameters as independent units, such as t-tests on speech rate, network comparison tests reveal differences in edge connections, node centrality, and overall connectivity between low- and high-anxiety states [44]. (3) Statistically robust edge estimation. Within a network, edges are usually statistically depicted using regularized partial correlations. These correlations, obtained after controlling for other variables and employing statistical regularization techniques, represent purer, more restrained, and interpretable connections among multivariate data [45]. By integrating these advantages, network analysis offers a holistic, data-driven framework that not only pinpoints “hub” speech parameters but also uncovers subtle systemic dysregulations that may be overlooked by traditional methods.

Therefore, this study aims to examine the complex impacts of the anxiety state on speech parameters using network analysis in a real stressed scenario. Specifically, the study has two primary objectives: (1) to investigate the complex associations between the anxiety state and speech parameters, with a focus on both direct and indirect relationships within the network; and (2) to explore how different levels of an anxiety state influence the patterns of associations between speech parameters.

Based on existing evidence showing that the anxiety state is associated with changes in vocal properties, we hypothesize that (1) speech parameters will exhibit a direct association with the anxiety state, and (2) higher levels of anxiety will fundamentally alter the network relationships among key speech parameters compared to lower anxiety states.

## 2. Materials and Methods

### 2.1. Settings and Participants

Participants were recruited from second-year undergraduate students at a large public university in Xi’an City, China. Participants were selected using a restricted homogeneous sampling method. Out of 569 students in the same grade, over 334 subjects were chosen based on their similar CET-4 test scores (a standardized English proficiency test in China; M = 499, SD = 32.85) to ensure comparable language proficiency across participants. A total of 316 valid participants were ultimately included in the analysis (191 males [60.4%] and 125 females [39.6%]). The age of participants ranged from 19 to 22 years old, with a mean age of 20.3 (±0.85) years. All participants provided written informed consent prior to participation.

The oral examination was conducted by course instructors as part of the curriculum. Following the image description task, participants were immediately instructed to self-rate their anxiety levels experienced during the task. The research assistants provided clear instructions, asking participants to “reflect on your feelings during the image description task and rate your anxiety level based on your immediate experience”. The self-rating was conducted using a 4-point scale (0 = no anxiety, 1 = mild anxiety, 2 = moderate anxiety, 3 = severe anxiety). This immediate assessment timing was crucial to capture the authentic anxiety-state levels while the experience was still fresh in participants’ memory, minimizing potential recall bias. Based on these ratings, participants were categorized into two groups: the low-anxiety group (ratings of 0–1; n = 119) and the high-anxiety group (ratings of 2–3; n = 197). This self-assessment approach aligns with prior research emphasizing the subjective nature of the anxiety state [46,47]. Following the assessment, the university counseling service were available for students who reported high anxiety levels. This study was conducted in accordance with the Declaration of Helsinki and received approval from the Ethics Committee of Xijing Hospital (KY20242053-C-1).

### 2.2. Materials and Procedure

This study utilized a cross-sectional design to explore differences in speech parameters between high- and low-anxiety individuals under a naturalistic stress-inducing condition. The anxiety-eliciting task was a foreign language oral examination, where participants were required to describe a thematic cartoon in English. This foreign language exam is an established ecological stressor widely recognized for its ability to provoke anxiety in second-language learners [48,49] because of the performance-related stress, fear of failing linguistic assessments, and anticipation of negative evaluations from proficient instructors [49,50,51]. The assessment consisted of a standardized picture description task featuring a thematic cartoon (topic: online and offline life) selected from a validated resource for formal language assessment materials. The instructions and interface of the online language testing platform are presented in Supplementary Figure S4. Previous psychophysiological studies have documented significant stress responses in such contexts, particularly among learners in high-stakes educational environments, with consistent elevations in both subjective anxiety measures and objective stress indicators [52,53].

Recordings were conducted in the university language labs with the ambient noise level maintained below 40 dB. The lab has a typical office acoustical setup with moderate reverberation time (approximately 0.4–0.5 s). Participants completed the oral exam task in a computer laboratory equipped with standardized workstations. Each participant was provided with a SANAKO SLH-07 headset featuring an integrated unidirectional microphone (frequency response: 40 Hz–16 kHz) to ensure consistent audio recording quality across all participants. The oral examination was conducted through an online English-language testing platform (https://www.tsinghuaelt.com/, (accessed on 18 January 2024)) designed for Chinese college curricula, with identical audio settings across all workstations. The microphone functionality was tested on the platform’s recording test page before beginning the oral exam (see Supplementary Figure S5).

The task instructions were presented uniformly through the testing platform interface. Students were required to talk about the picture displayed on the screen and were given in 45 s for preparation and 3 min to talk about it. After completing the task, participants were prompted to rate their anxiety state using the 4-point scale described earlier.

### 2.3. Speech Feature Extraction

Speech recordings were processed to extract five key acoustic parameters: jitter, fundamental frequency (F0), formants (F1/F2), intensity, and speech rate. These parameters were selected based on their established relevance to anxiety-related changes in speech [15,34,35].

The acoustic parameters analyzed in this study were defined as follows: Jitter refers to the cycle-to-cycle variation in fundamental frequency, which may indicate vocal instability and stress [23]. F0 reflects emotional arousal and tension [26]. F1/F2, the ratio of the first and second formants, represents interactions between pitch and vocal tract resonance and serves as a key indicator of articulatory precision and speech clarity [14]. Intensity captures the loudness of speech, measured in decibels (dB), and is often associated with vocal effort [54]. Speech rate measures the number of spoken syllables per second and reflects overall speech fluency and pacing [55].

Audio recordings were converted to a standardized format (16-bit WAV, 44.1 kHz) and trimmed to remove silence by Adobe Audition. Acoustic parameters were extracted using the open-source Python library Librosa (v0.10.0) and custom routines. Signals were pre-processed with a Hamming window (512 samples ≈ 23 ms at 22.05 kHz), 50% overlap (hop = 256 samples), and 0.97 pre-emphasis. F0 mean was extracted from indices 18/17 of the hsf array (Praat-aligned via Librosa). Formants F1/F2 were derived via 12th-order LPC analysis averaged across voiced segments using np.nanmean. Jitter followed the Praat equation. Intensity reflected RMS amplitude averaged over frames. Speech rate used Whisper (“base”) transcriptions.

### 2.4. Data Analysis

We utilized SPSS software (version 27.0) to perform the descriptive statistical analysis. An independent samples t-test was conducted to compare differences between the high- and low-anxiety groups regarding various acoustic parameters, including intensity, speech rate, F1/F2, F0, jitter, and state anxiety. Before constructing three networks (i.e., total, low-anxiety, and high-anxiety networks), the goldbricker function in the R package network tools was applied to determine the redundant nodes [56], with no potential node redundancy screened out in the three networks.

The three networks in this study were created using graphical Least Absolute Shrinkage and Selection Operator (gLASSO) combined with the Extended Bayesian Information Criterion (EBIC) [45]. Within each network, the edges reflect the partial (Spearman) correlation between two paired nodes, accounting for the influence of other nodes [45,57]. The gLASSO technique punishes small correlation coefficients to zero and results in a sparser and more interpretable network [45,58]. The EBIC tuning parameter (gamma) was adjusted to 0.5. The gamma controls the severity of the model selection, with higher values indicating that simpler models are preferred [45]. When gamma is set to 0.5, most spurious edges can be avoided [59]. Therefore, this more conservative approach makes the results of network structure more likely to be stable and reproducible. The EBICglasso function introduces gamma = 0.5 as “generally a good choice” and sets it as the default value [45,59]. The network visualization was generated using the Fruchterman–Reingold algorithm, through which nodes with weak and sparse connections are located on the periphery of the network, while nodes with strong and numerous connections tend to appear near the center of the network [60,61].

Expected influence, the sum value of all edges connecting to a specific node, was calculated for each node within low-anxiety and high-anxiety networks via R-package qgraph [61]. This indicator, when compared with the traditional centrality index (e.g., strength centrality), is more appropriate for networks that include both positive and negative edges [62]. Higher expected influence value of a node indicates its greater importance in the network [62,63].

To identify the accuracy of edge weights, a 95% confidence interval (1000 bootstrap samples) was plotted for each edge [64]. The stability of expected influence was determined by testing the correlation stability (CS) coefficient using the case-dropping bootstrap method (1000 bootstrap samples). The CS coefficient measures the maximum proportion of data that can be dropped to retain, with 95% certainty, a correlation of at least 0.7 with the centralities of the original network [64]. As recommended by Epskamp et al., it is considered ideal for the CS coefficient to be higher than 0.5 and not lower than 0.25 [64]. Additionally, we carried out bootstrapped difference tests (1000 bootstrap samples) for edge weights and expected influence. These analyses were performed using the R-package bootnet [64].

To investigate differences between low-anxiety and high-anxiety network characteristics, the R-package NetworkComparisonTest was employed with 1000 permutations [44]. We primarily concentrated on three key network characteristics: global expected influence, edge weights, and node expected influences. Given the exploratory nature of this study, no corrections were utilized for multiple comparisons [44,65].

## 3. Results

Table 1 presents the descriptive statistics for the anxiety state and acoustic parameters across the total sample, as well as the low- and high-anxiety groups. Self-reported anxiety differed significantly between groups, with the low-anxiety group reporting markedly lower levels compared to the high-anxiety group (*p* < 0.001, Cohen’s d = −3.731). This large effect size highlights a clinically meaningful distinction in perceived anxiety levels between the groups.

For acoustic parameters, jitter was slightly higher in the low-anxiety group compared to the high-anxiety group (*p* < 0.05, Cohen’s d = 0.229), and F0 was slightly lower in the low-anxiety group than in the high-anxiety group (*p* < 0.05, Cohen’s d = −0.232). Both differences were statistically significant but exhibited small effect sizes.

No statistically significant differences were observed for formant ratio (F1/F2), intensity, or speech rate (*p* > 0.2), and these variables demonstrated negligible effect sizes.

**Figure 1:**
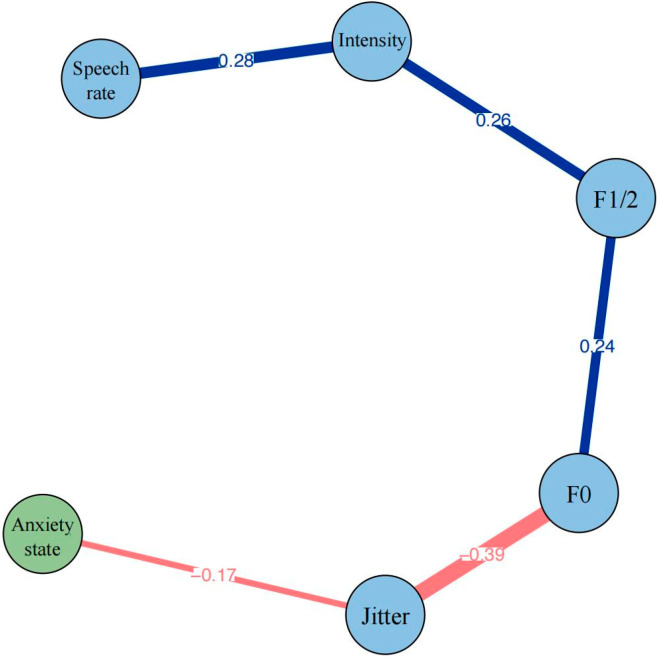
Network structure of anxiety state and speech indicators in total group. Blue edges represent positive correlations and red edges represent negative correlations. The thickness of the edge reflects the magnitude of the correlation. The value on the edge represents the edge weights of two corresponding nodes.

Figure 1 illustrates the network structure of the anxiety state and various speech-related indicators in the total group. It is evident that the anxiety state is negatively connected to only one speech indicator—jitter (edge weight = −0.17). The connections between the speech indicators themselves are generally strong and predominantly positive. Among the speech indicators, three positive connections are, respectively, speech rate–intensity (edge weight = 0.28), intensity–F1/2 (edge weight = 0.26), and F1/2–F0 (edge weight = 0.24). There exists only one strong negative connection between jitter and F0 (edge weight = −0.39). The bootstrapped 95% confidence interval indicates that the edge weights are relatively accurate (Figure S1a in the Supplementary Materials). Figure S1b shows the difference test results of all edges.

**Figure 2:**
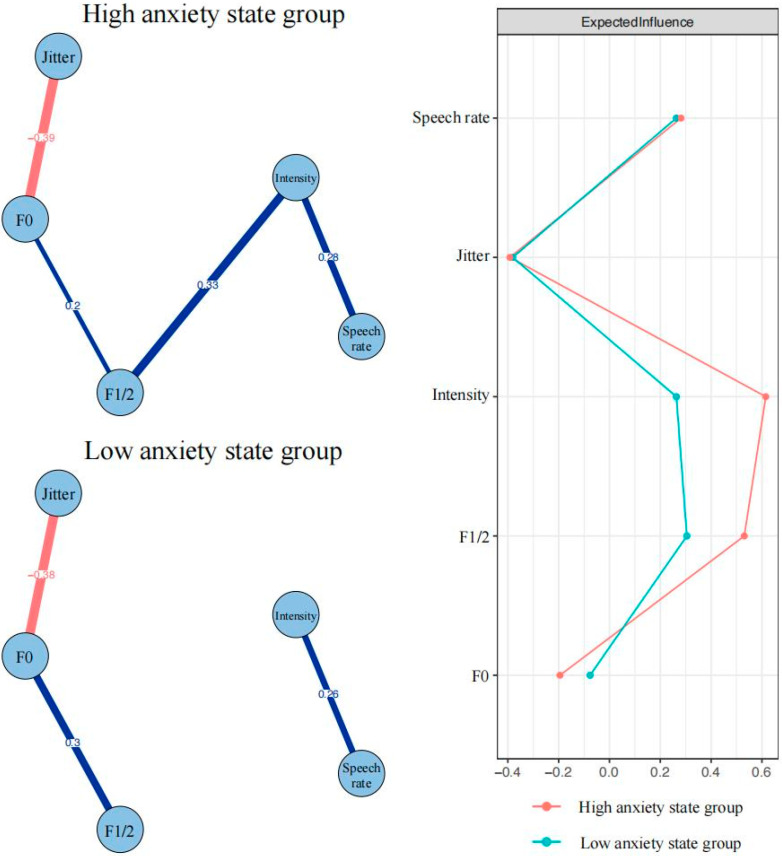
Speech indicator networks in high- and low-anxiety-state groups using average layout and bridge centrality plots. Blue edges represent positive correlations and red edges represent negative correlations. The thickness of the edge reflects the magnitude of the correlation. The value on the edge represents the edge weights of two corresponding nodes.

In the speech indicator network of the high-anxiety-state group (see upper left of Figure 2), the pattern of connections between speech indicators is very similar to that in Figure 1. Three positive connections are, respectively, speech rate–intensity (edge weight = 0.28), intensity–F1/2 (edge weight = 0.33), and F1/2–F0 (edge weight = 0.20). Only one strong negative connection is between jitter and F0 (edge weight = −0.39). In the speech indicator network in the low-anxiety-state group (see lower left of Figure 2), except for the absence of a connection between intensity and F1/2, the connection patterns between the other indicators remain similar to the high-anxiety group. Bootstrapped 95% confidence intervals indicate that the edge weights in the two samples are relatively accurate (Figure S2a,c in the Supplementary Materials). Figure S2b,d show the edge weights’ difference test results.

The expected influence results for the high-anxiety- and low-anxiety-state groups are plotted in the right section of Figure 2. In the high-anxiety-state network, intensity has the strongest expected influence (raw value = 0.62). In the low-anxiety-state network, F1/2 has the strongest expected influence (raw value = 0.30). The CS coefficients of expected influences for high-anxiety-state and low-anxiety-state networks are 0.67 and 0.36, respectively (Figure S3a,c in the Supplementary Materials). Figure S3b,d show the nodes expected influences’ difference test results.

The results of the network comparison test reveal that only one connection among speech indicators exhibit significant differences: F1/2-intensity (*p* = 0.02). The node expected influences of two groups show no significant differences. In addition, the global expected influence of two groups also show no significant differences (high-anxiety-state group = 0.42; low-anxiety-state group = 0.19; S = 0.23, *p* = 0.43).

## 4. Discussion

This study employed network analysis to explore the impacts of anxiety state on speech parameters in ecologically valid settings. Two distinct networks were constructed. The first network elucidated the relationships between anxiety state and speech parameters, with particular focus on the direct associations and transmission effects between parameters. The second network identified how different levels of anxiety state influence the association patterns among speech parameters.

From a global view, the network structure demonstrated a clear, chain-like arrangement of speech parameters, with jitter emerging as the only parameter directly connected to the anxiety state. This finding may suggest the role of jitter as the most critical acoustic indicator of anxiety responses under stress. Specifically, the correlation between the anxiety state and jitter indicates that increased anxiety levels may disturb the vocal stability and trigger alterations in pitch. This finding aligns with previous research suggesting that anxiety-induced physiological changes affect laryngeal muscle tension and respiratory control [66]. Notably, the effect of anxiety on jitter is not universally observed. Some studies have documented increased jitter in response to stress [9,14,67], often attributed to heightened laryngeal and cricothyroid tension leading to less stable vocal fold vibration. Other research, however, includes findings similar to ours, where jitter decreases under real-world stress conditions such as exam or emergencies [37,68]. The reduced jitter in these scenarios could be justified by the cognitive load and emotional load induced alone or in combination [35]. And it could also be attributed to the compensatory vocal mechanisms under anxiety conditions, suggesting heightened psychological arousal may lead to increased laryngeal muscle tension, consequently leading to more regular vocal fold vibration. Alternatively, given the dynamic nature of anxiety during a speech task, speakers may experience peak anxiety at the beginning of a task, which may gradually diminish as they adapt to the stressor over time. This temporal variation in anxiety could influence jitter differently at different phases of the task. As such, if jitter reduction is driven by compensatory mechanisms, this effect may emerge later in the task as speakers adapt to the stressors.

From the perspective of individual differences, these contradictory patterns may reflect variability in speech motor control or baseline vocal tension. When in a stressed state, jitter could be caused by either small variations or asymmetries in the cricothyroid muscle tension [69] and/or fluctuations in subglottal pressure [70] and/or perturbations in the mucous of the vocal folds [71]. Speakers with greater baseline vocal tension may show a decrease in jitter as a form of physiologically “locked” vocal fold vibration, whereas those with lower baseline tension might exhibit an increase in jitter when confronted with stress. So, baseline vocal tension could be measured in the future by surface electromyography of the vocal tract, or general speech motor control could be pre-assessed to elucidate the individual differences in jitter responses under anxiety.

Most importantly, our research extends beyond the confines of the lab, offering greater ecological validity by examining stress responses in more naturalistic settings. Previous network analysis study conducted in a controlled laboratory setting identified jitter as the only speech parameter directly connected to self-reported negative affect [14]. Consistently, we also found jitter to be the only speech parameter directly linked to stress responses, reinforcing its potential as a robust acoustic biomarker of stress. These findings suggest that jitter holds significant promise as a reliable indicator of stress whether in controlled experimental environments or in daily life.

Furthermore, jitter also correlates inversely with F0, possibly acting as a “bridge” variable in transmitting anxiety effects onto other vocal parameters. A direct connection with F0 was expected, as jitter by definition refers to the variations that occur in F0. Previous research mostly showed that increased fundamental frequency (F0) is accompanied by elevated cycle-to-cycle frequency perturbation (jitter) under stress conditions [37,72,73]. As Giddens et al. reported, increased vocal fold tension under stress typically raises F0 while also leading to more irregular vocal fold vibration patterns, resulting in increased jitter [15]. Interestingly, our finding implies that despite the stress-induced elevation in pitch, speakers maintain or even enhance their vocal stability, possibly as a physiological adaptation to maintain speech clarity under pressure. Together, this sequential organization suggests a hierarchical relationship in how anxiety manifests through various vocal parameters and potentially reflects the intricate mechanisms underlying speech production under stress.

The total group network analysis also showed a positive interplay among core speech parameters, where F0, formant frequencies (F1/2), intensity, and speech rate exhibited positive interconnections, suggesting a coherent vocal pattern that might collectively shift when speaking under challenging conditions. From a physiological standpoint, as F0 rises, speakers often inadvertently increase the intensity and modify the articulation captured by F1/2, which can also interact with the rate of delivery. Specifically, the positive correlation between F0 and F1/2 suggests that as speakers raise their pitch under stress, they also exhibit higher formant frequencies. This relationship likely reflects complex changes in vocal tract configuration under stress, as documented by Scherer [74] and Murray et al. [75]. The positive relationship between F1/2 and intensity indicates that speakers who produce higher formant frequencies also tend to speak louder under stress, a pattern consistent with acoustic theory and supported by studies of stress-induced vocal changes, noting that stress-induced physiological changes often result in simultaneous increases in vocal intensity and articulatory effort [26]. The moderate positive correlation between intensity and speech rate further highlights the possible activation effect, wherein speakers under stress tend to speak both louder and slightly faster. Stress-induced arousal typically results in increased respiratory drive and heightened vocal cord tension, which can raise vocal intensity while also promoting a faster articulation tempo, as supported by studies on emotional vocal expression [26,74]. This relationship is consistent with findings that stress can amplify vocal energy and motor coordination, driving dynamic changes across vocal parameters. Moreover, the observed interconnections among F0, F1/2, intensity, and speech rate align with Scherer’s push effect theory [74], in which physiological arousal during emotional states initiates coordinated adaptations across multiple vocal subsystems. These speech changes arise from increased sympathetic nervous system activation, subsequently affecting breathing patterns, vocal fold tension, and articulatory precision, eventually leading to synchronized shifts in pitch, loudness, and speech pacing. Thus, this chain of relationships reflects how stress modifies vocal behavior holistically.

Most interestingly, the network analysis revealed distinct patterns of acoustic parameter connections between high- and low-anxiety groups, indicating that anxiety may fundamentally restructure the relationships among vocal parameters under stress. Similar to the total group network, the high-anxiety network exhibited greater association among acoustic parameters, with multiple strong connections forming a chain-like structure. This increased parameter coupling in the high-anxiety group aligns with Scherer’s component process model [76] and Goberman et al.’s stress-induced vocal synchronization [23], suggesting that tighter coupling among acoustic parameters may serve as reliable biomarkers for anxiety states. The low-anxiety network displayed a sparse structure with fewer strong connections between parameters, which may suggest more relaxed vocal control compared to high-anxiety states [35]. This finding is consistent with previous research showing that neutral or low-arousal states typically demonstrate more independent variation in acoustic features [26]. This separation between intensity and F1/2 in the low-anxiety network indicates that speakers could maintain their natural articulation without intensity compensation, in contrast to the strong connection of F1/2 and intensity in the high-anxiety group, where speakers need to enhance their vocal intensity to compensate for the weak articulation. This pattern likely reflects what Lindblom termed “hypo-speech”, where speakers optimize articulatory effort to balance communicative efficiency and minimal physiological cost within a given context [77]. Thus, these contrasting networks offer valuable diagnostic potential for anxiety detection. The presence or absence of intensity–articulation coupling may serve as a reliable marker for anxiety states, particularly in ecologically valid settings.

The expected influence analysis provides additional insights into the relative importance of different speech parameters within each anxiety-state network. In the high-anxiety state, intensity (EI = 0.62) emerged as the most central node, suggesting the dominant role of intensity in the high-anxiety group, which reflects the compensatory vocal effort to maintain clarity despite stress-induced physiological arousal [74]. Conversely, in the low-anxiety state, F1/F2 (EI = 0.30) showed the strongest influence, indicating greater articulatory freedom when cognitive resources are not consumed by anxiety. This pattern aligns with Lindblom’s hyper- and hypo-speech framework, suggesting that speakers optimize vocal effort relative to communicative demands [77]. Comparing the expected influence between two groups, the low-anxiety-state network displayed more evenly distributed expected influence values across F1/2, intensity, and speech rate. This balanced distribution may suggest that parameters operate with greater independence rather than being dominated by specific features. The difference in expected influence between anxiety states further supports our earlier observations about the distinctive network organizations under different anxiety conditions. However, the absence of significant differences in nodes’ expected influence and global expected influence between groups suggests that while the organization of speech parameters differs between anxiety states, the overall influence structure remains relatively stable.

It is also worth noting that the use of English as a non-native language in our task likely amplified the anxiety triggered by the exam through foreign language anxiety (FLA) mechanisms [78], particularly affecting articulatory precision (F1/F2) and vocal effort (intensity) [79,80]. This aligns with FLA research showing that linguistic insecurity exacerbates vocal changes [81,82]. Non-native speakers often exhibit reduced articulatory control and increased vocal tension due to unfamiliar phonemes [83,84], which may explain the strong intensity–F1/F2 coupling observed in our high-anxiety network. However, the stability of jitter as a biomarker across anxiety states suggests some vocal stress responses transcend language-specific factors.

Previous research on speech biomarkers primarily focused on evaluating individual acoustic parameters in isolation. However, our study indicates the importance of the relationships between these parameters as additional potential biomarkers, providing a fresh perspective on stress-related vocal dynamics. This shift broadens the scope of traditional biomarker research by emphasizing the need to examine how vocal features interact within a speech parameter network rather than concentrating solely on individual values.

This study first employs network analysis of vocal parameters in an ecologically valid anxiety context, filling the gap in the existing anxiety research that has largely been restricted to laboratory environments. In addressing the first objective, we uncovered a network across anxiety state and speech parameters, which highlights the direct associations between jitter and anxiety state, and transmission effects among vocal features. Importantly, jitter is found to be the sole speech parameter consistently linked to the anxiety state across all networks, underscoring its potential as a robust acoustic biomarker for stress. For the second objective, we demonstrated that varying levels of the anxiety state change the association patterns among speech features fundamentally, manifesting as the absence of an intensity and F1/F2 connection. These findings could enhance voice-based anxiety screening tools by prioritizing jitter and intensity–F1/F2 coupling as biomarkers. For instance, mobile apps analyzing speech during telehealth consultations or workplace stress assessments could flag high-risk states in real time. Clinically, this could supplement traditional surveys with objective vocal metrics.

Several methodological limitations warrant consideration. First, by recruiting mainly non-native English speakers from a single university, our findings may not fully generalize to other linguistic or cultural settings. Second, we relied on a single speech task (picture description), which may not capture the full range of anxiety-inducing scenarios—such as public speaking or interpersonal conflict. Furthermore, our binary categorization of anxiety (low vs. high) could obscure more nuanced or non-linear trajectories, including the possibility that jitter initially increases at moderate anxiety levels but later stabilizes as speakers adapt. Additionally, anxiety was measured solely via self-report, and the absence of physiological indicators (e.g., cortisol, heart rate variability) may limit the objectivity of anxiety assessment. Finally, to keep computational demands manageable, we focused on a limited set of vocal parameters, potentially missing other relevant acoustic features.

Therefore, future research should recruit participants from diverse populations to broaden the applicability of the findings. Studies should also examine additional stress-inducing contexts and analyze speech production at multiple time points to better capture the dynamic progression of anxiety and the potential emergence of compensatory mechanisms. Furthermore, regression modeling of anxiety could be incorporated to determine the trend of jitter response. Further, incorporating physiological indicators and additional acoustic parameters, such as the harmonics-to-noise ratio, could be used to examine detailed anxiety-related vocal changes. Finally, individual differences in speech motor control and baseline anxiety should be explored to uncover the mechanisms linking anxiety with vocal parameters.

## 5. Conclusions

This study provides insights into the relationship between anxiety states and speech parameters using network analysis in an ecologically valid setting. By examining the interconnections among vocal features, we identified jitter as the sole speech parameter consistently linked to the anxiety state across all networks, underscoring its potential as a robust acoustic biomarker for stress. Additionally, the coupling between intensity and F1/F2 in high-anxiety states highlights how anxiety fundamentally reorganizes vocal parameter relationships, reflecting compensatory mechanisms under stress. Our findings contribute to a more comprehensive understanding of how anxiety manifests through interconnected vocal parameters. We also demonstrate that the ecological validity of using real-world stressors, such as oral examinations, could capture authentic anxiety responses, which enhances the applicability of our results to real-life contexts. These findings have practical implications for developing automated, speech-based anxiety screening tools that prioritize jitter and intensity–F1/F2 coupling as key markers. This study may offer a fresh perspective for developing objective, non-invasive assessments of anxiety.

## Figures and Tables

**Table 1:** Anxiety state and acoustic variables across total, low-anxiety, and high-anxiety groups.

Variables	Total Group (Mean ± SD)	Low-Anxiety Group (Mean ± SD)	High-Anxiety Group (Mean ± SD)	Low-Anxiety Group vs. High-Anxiety Group (*p*-Value)	Cohen’s d
Anxiety state	1.83 ± 1.01	0.70 ± 0.46	2.51 ± 0.50	0.000 **	−3.731
Jitter	0.50 ± 0.07	0.51 ± 0.06	0.49 ± 0.08	0.040 *	0.229
F0	181.88 ± 18.53	179.20 ± 18.70	183.49 ± 18.29	0.046 *	−0.232
F1/F2	0.25 ± 0.05	0.25 ± 0.06	0.25 ± 0.05	0.212	0.145
Intensity	67.54 ± 5.64	67.78 ± 5.41	67.40 ± 5.78	0.561	0.068
Speech rate	1.26 ± 0.57	1.26 ± 0.61	1.26 ± 0.55	0.999	0.000

Variables of Significance:* *p* ≤ 0.05, ** *p* ≤ 0.001.

## Data Availability

The data presented in this study are available on request from the corresponding author. The data are not publicly available due to ethical restrictions.

## Supplementary Materials

The following supporting information can be downloaded at: https://www.mdpi.com/article/10.3390/brainsci15030262/s1, Figure S1: Edge weights’ accuracy and difference test results of the anxiety state and speech indicators network in total group; Figure S2: Edge weights’ accuracy and difference test results of the speech indicators networks in high- and low-anxiety-state groups; Figure S3: Node expected influences’ stability and difference test results of the speech indicators networks in high- and low-anxiety-state groupss; Figure S4: Screenshot of the Online Language Testing Platform Interface; Figure S5: Recording Test Page of the Online Language Testing Platform; Figure S6: Experimental Setup for Data Collection; Table S1: Edge invariance test results of the networks in high- and low-anxiety state group.
