# Supplementary material for: How Anxiety State Influences Speech Parameters: A Network Analysis Study from a Real Stressed Scenario
Source: Brain Sci. 2025 Feb 28;15(3):262. doi: 10.3390/brainsci15030262 (PMC11939969; doi:10.3390/brainsci15030262)
Supplement: Supplementary file 1 [file brainsci-15-00262-s001.zip › brainsci-3483595-supplementary.pdf]

Supplementary Materials

1. Figure S1. Edge weights' accuracy and difference test results of the anxiety state and speech indicators network in the total group.
2. Figure S2. Edge weights' accuracy and difference test results of the speech indicators networks in high- and low-anxiety state groups.
3. Figure S3. Node expected influences' stability and difference test results of the speech indicators networks in high- and low-anxiety state groups.
4. Table S1. Edge invariance test results of the networks in high- and low-anxiety state groups.
5. Figure S4. Screenshot of the Online Language Testing Platform Interface.
6. Figure S5. Recording Test Page of the Online Language Testing Platform.
7. Figure S6. Experimental Setup for Data Collection.

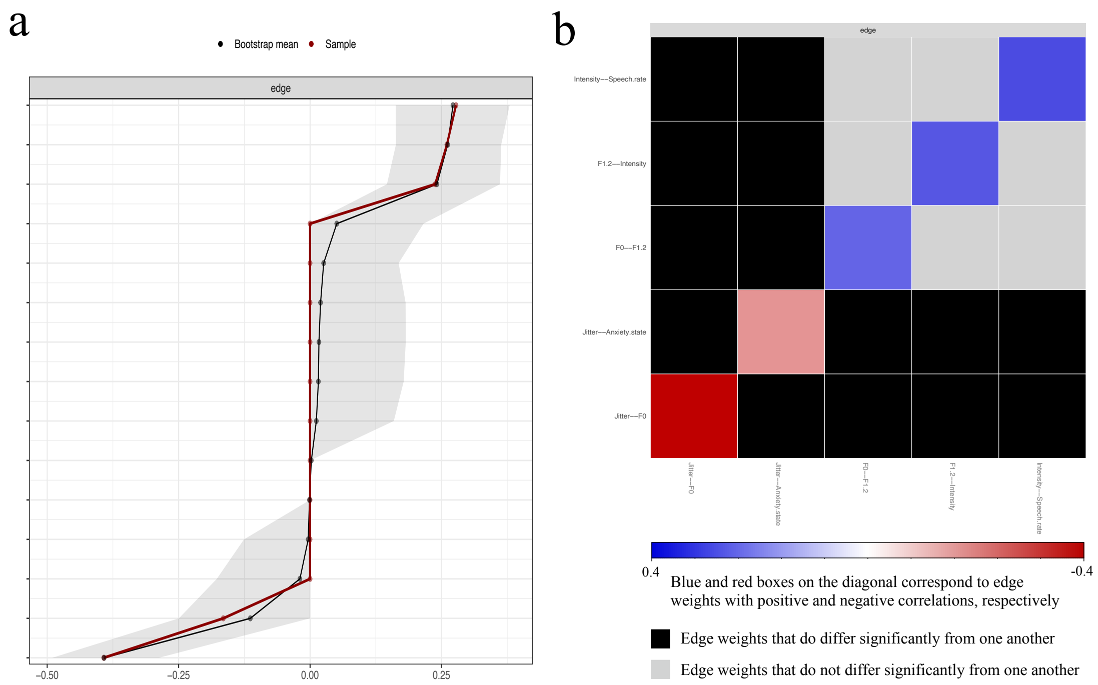

Figure S1. Edge weights' accuracy and difference test results of the anxiety state and speech indicators network in total group

Note: (a) Accuracy of edge weights. The red line depicts the sample edge weights and the gray bar depicts the bootstrapped confidence interval. (b) Bootstrapped difference test for edge weights. Gray boxes indicate edge weights that do not differ significantly from one another, while black boxes indicate edge weights that do differ significantly. Blue and red boxes on the diagonal correspond to edge weights with positive and negative correlations, respectively.

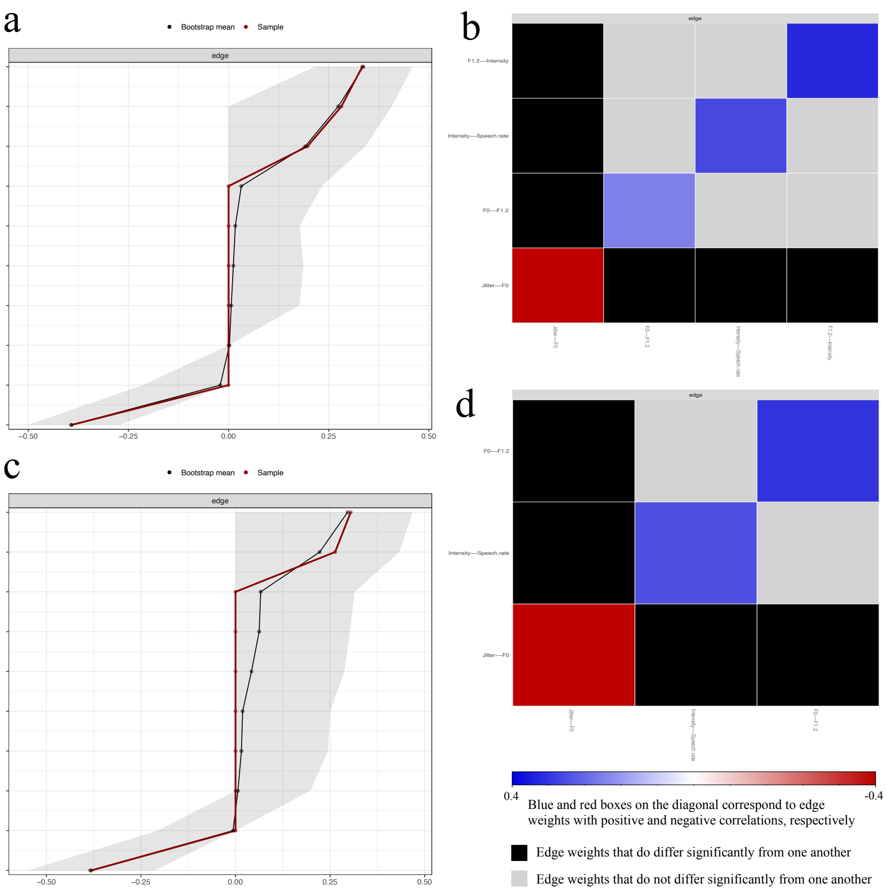

Figure S2. Edge weights' accuracy and difference test results of the speech indicators networks in high and low anxiety state groups

Note: (a) Accuracy of edge weights of the speech indicators network in high anxiety state group. The red line depicts the sample edge weights and the gray bar depicts the bootstrapped confidence interval. (b) Bootstrapped difference test for edge weights of the speech indicators network in high anxiety state group. Gray boxes indicate edge weights that do not differ significantly from one another, while black boxes indicate edge weights that do differ significantly. Blue and red boxes on the diagonal correspond to edge weights with positive and negative correlations, respectively. (c) Accuracy of edge weights of the speech indicators network in low anxiety state group. The red line depicts the sample edge weights and the gray bar depicts the bootstrapped confidence interval. (d) Bootstrapped difference test for edge weights of the speech indicators network in low anxiety state group. Gray boxes indicate edge weights that do not differ significantly from one another, while black boxes indicate edge weights that do differ significantly. Blue and red boxes on the diagonal correspond to edge weights with positive and negative correlations, respectively.

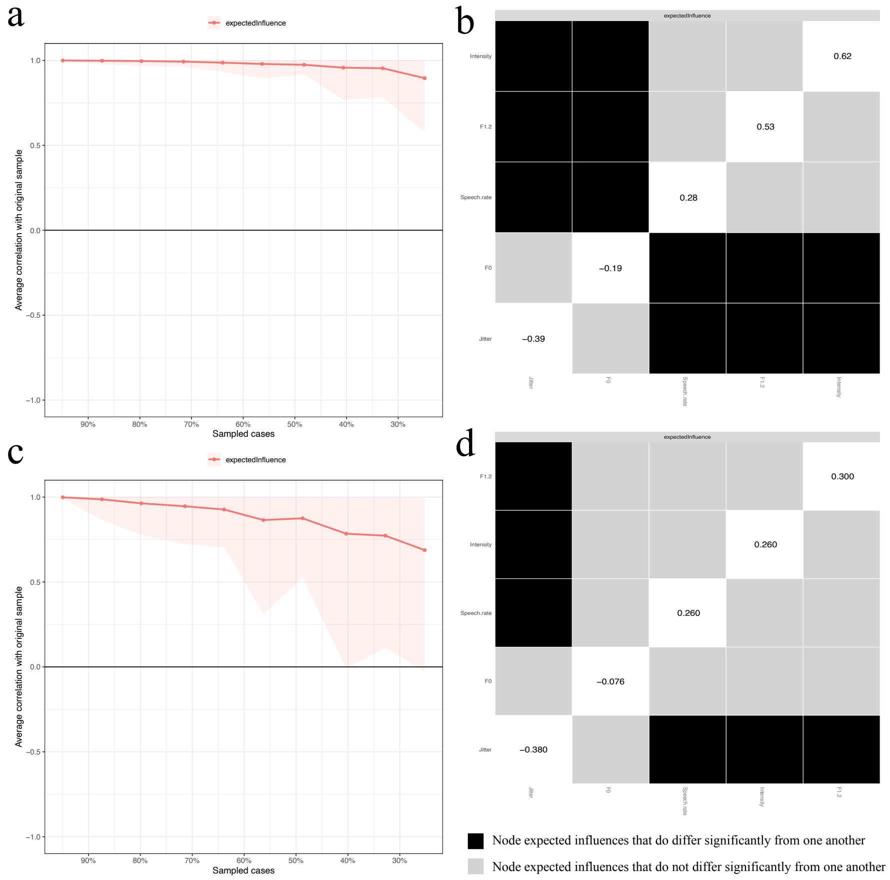

Figure S3. Node expected influences' stability and difference test results of the speech indicators networks in high and low anxiety state groups

Note: (a) Stability of the expected influence of the speech indicators network in high anxiety state group. The red bar represents the average correlation between expected influence. (b) Bootstrapped difference test for the expected influence of the speech indicators network in high anxiety state group. Gray boxes indicate the expected influence that do not differ significantly from one another, while black boxes indicate the expected influence that do differ significantly. The number in the white boxes (i.e., diagonal line) represent the value of the expected influence. (c) Stability of the expected influence of the speech indicators network in low anxiety state group. The red bar represents the average correlation between expected influence. (d) Bootstrapped difference test for the expected influence of the speech indicators network in low anxiety state group. Gray boxes indicate the expected influence that do not differ significantly from one another, while black boxes indicate the expected influence that do differ significantly. The number in the white boxes (i.e., diagonal line) represent the value of the expected influence.

Table S1. Edge invariance test results of two networks in high and low anxiety state groups

Edge	Test statistic E	p-value
Jitter-F0	0.01	0.91
Jitter-F1/2	0.00	1.00
F0-F1/2	0.11	0.36
Jitter-Intensity	0.00	1.00
F0-Intensity	0.00	1.00
F1/2-Intensity	0.33	0.02
Jitter-Speech rate	0.00	1.00
F0-Speech rate	0.00	1.00
F1/2-Speech rate	0.00	1.00
Intensity-Speech rate	0.02	0.88

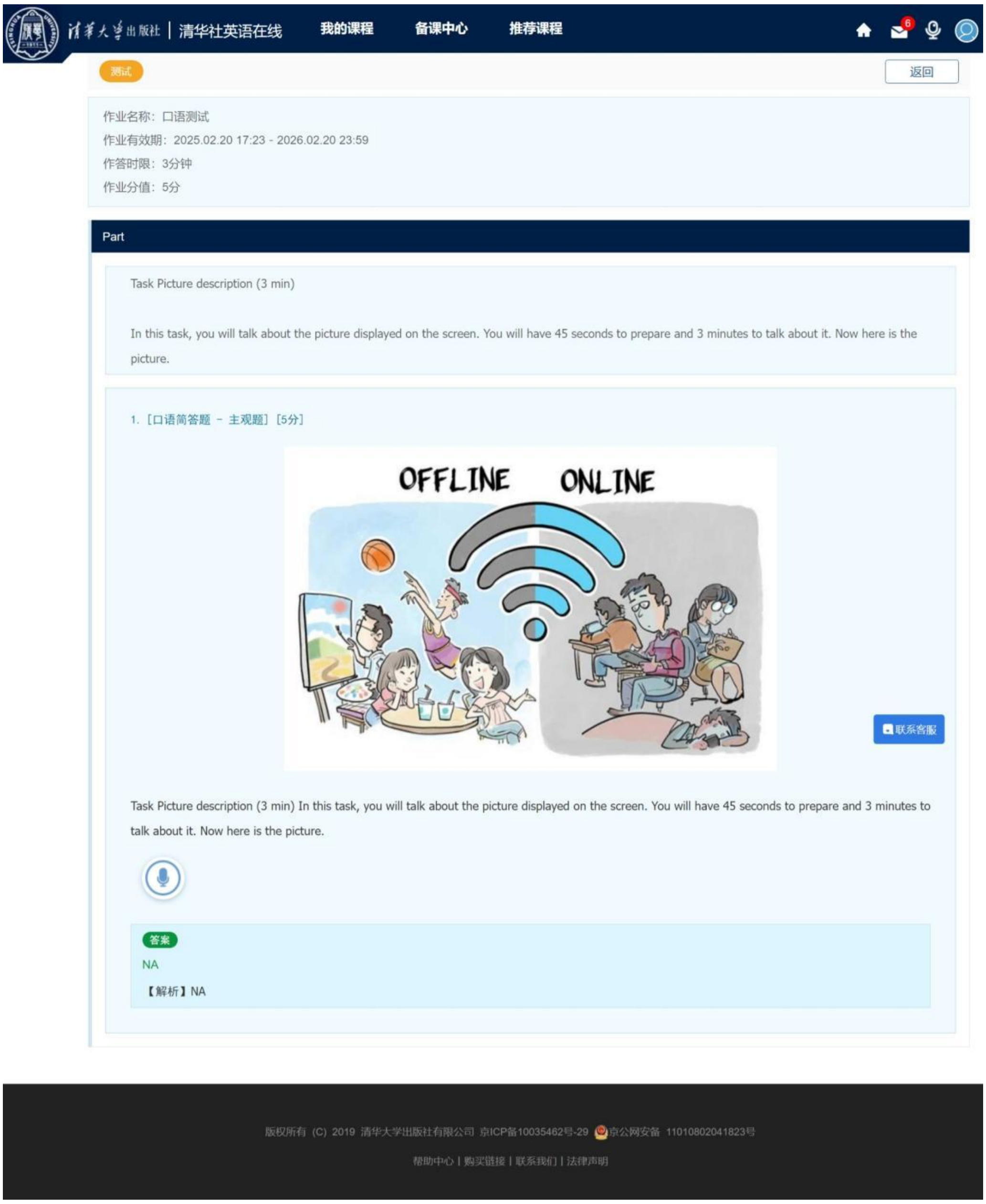

Figure S4. Screenshot of the Online Language Testing Platform Interface

Note: Supplementary Figure S1. This figure provides a screenshot of the online platform used for the oral examination task. The platform interface displays the task instructions, a cartoon prompt for description in English, and a timer. The task required participants to prepare for 45 seconds and then describe the cartoon within 3 minutes. This interface was standardized for all participants to ensure consistency in task administration.

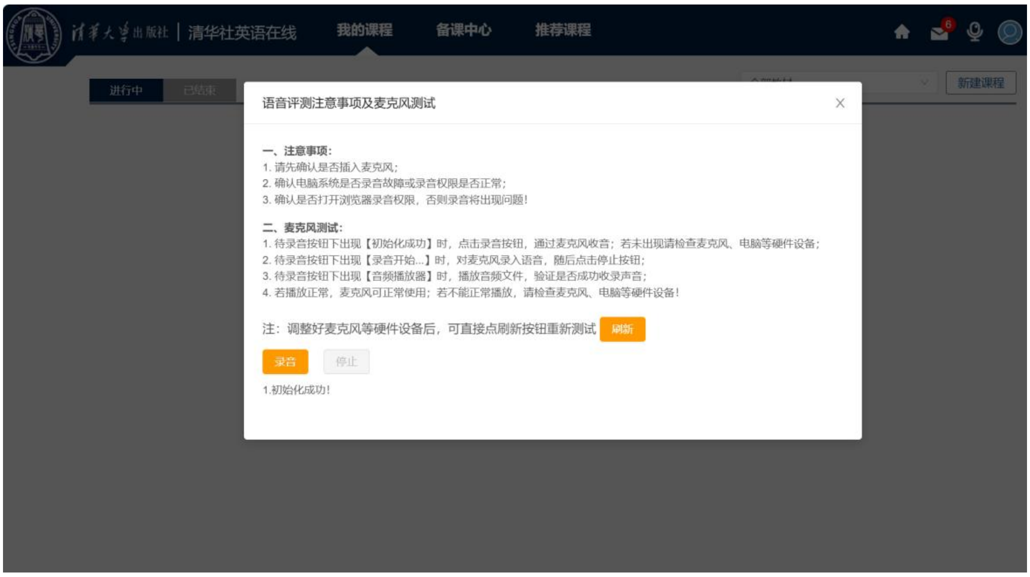

Figure S5. Recording Test Page of the Online Language Testing Platform

This figure shows the recording test page from the platform used for the oral exam. Participants were required to test microphone functionality and ensure correct hardware setup before proceeding with the oral exam task. The instructions emphasized technical readiness, which was standardized for all participants to prevent confounding factors during data collection.

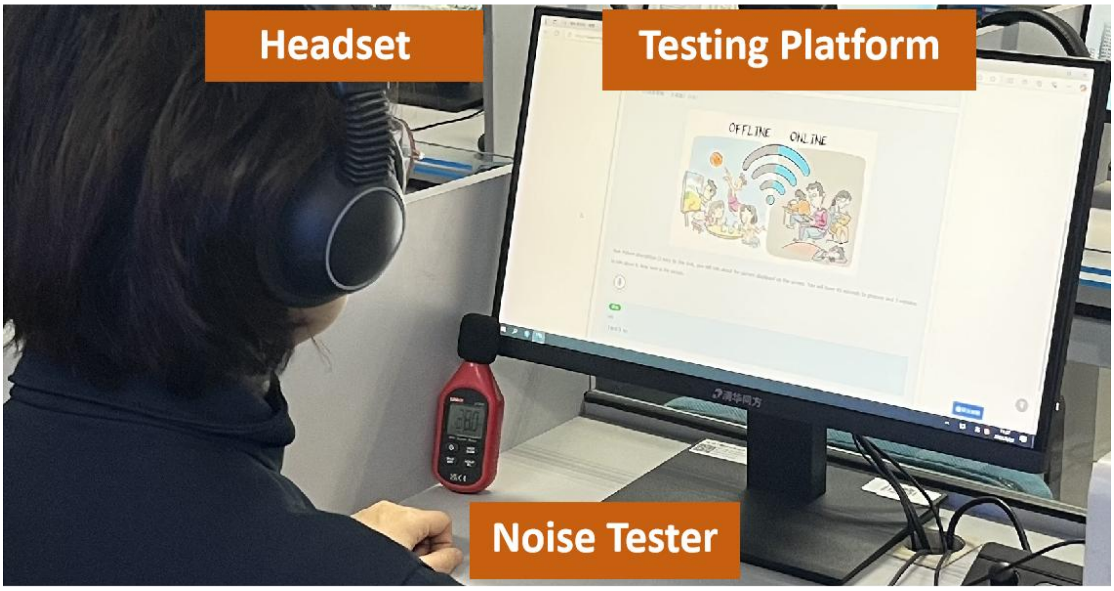

Figure S6. Experimental Setup for Data Collection

The experimental setup includes a participant seated at a computer interface while wearing a headset for audio output and equipped with a noise tester to monitor sound levels in the environment. The setup was designed to ensure controlled conditions for speech task performance and data recording.
